# Influence of Two Major *Toxoplasma Gondii* Virulence Factors (ROP16 and ROP18) on the Immune Response of Peripheral Blood Mononuclear Cells to Human Toxoplasmosis Infection

**DOI:** 10.3389/fcimb.2019.00413

**Published:** 2019-12-04

**Authors:** Alejandro Hernández-de-los-Ríos, Mateo Murillo-Leon, Luz Eliana Mantilla-Muriel, Ailan Farid Arenas, Mónica Vargas-Montes, Néstor Cardona, Alejandra de-la-Torre, Juan Carlos Sepúlveda-Arias, Jorge Enrique Gómez-Marín

**Affiliations:** ^1^Grupo de Estudio en Parasitología Molecular (GEPAMOL), Facultad de Ciencias de la Salud, Centro de Investigaciones Biomédicas, Universidad del Quindío, Armenia, Colombia; ^2^Grupo Infección e Inmunidad, Facultad de Ciencias de la Salud, Universidad Tecnológica de Pereira, Pereira, Colombia; ^3^Universidad Antonio Nariño, Armenia, Colombia; ^4^Grupo NeURos, Unidad de Inmunología, Escuela de Medicina y Ciencias de la Salud, Universidad del Rosario, Bogota, Colombia

**Keywords:** peripheral blood mononuclear cells, *Toxoplasma*, ROP16 protein, ROP18 protein, ocular toxoplasmosis, cytokines, polymorphisms

## Abstract

*Toxoplasma gondii* ROP16 and ROP18 proteins have been identified as important virulence factors for this parasite. Here, we describe the effect of ROP16 and ROP18 proteins on peripheral blood mononuclear cells (PBMCs) from individuals with different clinical status of infection. We evaluated IFN-γ, IL-10, and IL-1β levels in supernatants from PBMCs cultures infected with tachyzoites of the *T. gondii* wild-type RH strain or with knock-out mutants of the *rop16* and *rop18* encoding genes (RHΔ*rop16* and RHΔ*rop18*). Cytokine secretion was compared between PBMCs obtained from seronegative individuals (*n* = 10), with those with chronic asymptomatic (*n* = 8), or ocular infection (*n* = 12). We also evaluated if polymorphisms in the genes encoding for *IFN*-γ, *IL-10, IL-1*β, Toll-like receptor 9 (*TLR9*), and purinoreceptor *P2RX7* influenced the production of the encoded proteins after *ex vivo* stimulation. In individuals with chronic asymptomatic infection, only a moderate effect on IL-10 levels was observed when PBMCs were infected with RHΔ*rop16*, whereas a significant difference in the levels of inflammatory cytokines IFN-γ and IL-1β was observed in seronegative individuals, but this was also dependent on the host's cytokine gene polymorphisms. Infection with ROP16-deficient parasites had a significant effect on IFN-γ production in previously non-infected individuals, suggesting that ROP16 which is considered as a virulence factor plays a role during the primary infection in humans, but not in the secondary immune response.

## Introduction

*Toxoplasma gondii* is an obligate intracellular parasite that infects a broad range of vertebrate hosts. In humans, the most important clinical manifestations are as follows: (*i*) retinochoroiditis, being the most important cause of posterior uveitis and an important cause of blindness in certain countries (De-la-Torre et al., 2009), (*ii*) congenital toxoplasmosis, a public health problem responsible for early childhood morbidity and mortality (Gómez-Marin et al., 2011), and (*iii*) cerebral toxoplasmosis, the most important cause of neurological symptoms in HIV-infected patients (Cardona et al., 2011). There are host and parasite factors that contribute to the clinical outcome of the infection. One of the most extensively studied are ROP proteins, produced by a set of specialized secretory organelles in the parasite called the rhoptries.

In murine models, quantitative trait locus analysis has allowed the identification of genes that contribute to differences between virulent and non-virulent strains of the parasite (Saeij et al., 2006; Taylor et al., 2006). These genes, which encode the polymorphic serine/threonine (S/T) protein kinases secreted by the rhoptries, are also called ROP kinases proteins (Peixoto et al., 2010). Two of the most extensively studied are ROP16 (TGME49_062730) and ROP18 (TGME49_005250) kinases. ROP16 phosphorylates STAT3 (Ong et al., 2010) and STAT6 (Yamamoto et al., 2009) transcription factors, thereby leading to altered cytokine profiles and the repression of IL-12 signaling (Saeij et al., 2007) required for the generation of IFN-γ by CD4+ and CD8+ T lymphocytes. Both, IL-12 and IFN-γ production are essential for the host to survive infection with *T. gondii* (Scharton-Kersten et al., 1996), and the control of these proinflammatory mediators is achieved by the induction of anti-inflammatory cytokines such as IL-10 (Denkers et al., 2012). On the other hand, in mice, ROP18 interferes with the function of host immunity related GTPases by phosphorylating these proteins thus, avoiding their interaction with the parasitophorous vacuole membrane (Steinfeldt et al., 2010). Although the discovery of these virulence factors in mice prompted an explosion of work to reveal the mechanisms underlying parasite virulence, there are only a few reports on the possible roles of these genes in the human immune response against the parasite (Niedelman et al., 2012; Portillo et al., 2017). Therefore, the aim of this study was to evaluate the secretion of IFN-γ, IL-10, and IL-1β in PBMCs from individuals with different clinical status of infection (ocular, chronic asymptomatic, and non-infected) when stimulated with the virulent wild-type (WT) *T. gondii* RH strain, and with knock-out (KO) *T. gondii rop16* and *rop18* mutants.

## Materials and Methods

### Ethical Considerations

This study was conducted according to the tenets of the Declaration of Helsinki, and strictly adhered to the Guide for Good Laboratory Procedures. It was approved by the Ethics Committee of the Universidad del Quindío, Colombia. All patients agreed to participate in the study and signed the informed consent according to the *Minsalud 8430-resolution*. The results of pertinent clinical laboratory analysis were given to the patient and the attending physician.

### Sample Population

Peripheral blood samples were collected from 12 patients with ocular toxoplasmosis (OT), 8 with chronic asymptomatic infection (Asym), and 10 individuals seronegative for IgG and IgM anti-*Toxoplasma* antibodies (Neg) who agreed to participated in this study. Patients with OT were recruited during ophthalmological consultation at the Universidad del Quindío. The clinical diagnosis of OT was based on criteria previously described (De-la-Torre et al., 2009). Briefly, active OT was defined by the presence of an active creamy-white focal retinal lesion, which eventually resulted in hyperpigmented retinochoroidal scars in either eye. Central lesions were defined as lesions located within the large vascular arcades. Lesion sizes were measured in disk diameters, and the inflammation intensity in the anterior segment was measured by counting the number of cells in the anterior chamber using biomicroscopy, and in the posterior pole also by visualizing the vitreous haze using fundoscopy. The inflammation grade was registered according to the standardization of uveitis nomenclature for the reporting of clinical data (Jabs et al., 2005). When the lesions were inactive, the results of the last inflammatory period were recorded from the clinical charts. Asymptomatic patients that agreed to participate had a serological status of chronic infection (IgG anti-*Toxoplasma* positive and IgM anti-*Toxoplasma* negative) and a fundoscopic eye examination negative for ocular lesions.

### Parasites

The WT *T. gondii* strain RH or ROP16 and ROP18 null mutants (RHΔ*rop16* and RHΔ*rop18*) tachyzoites were maintained by serial passes in confluent monolayers of human foreskin fibroblast (HFF, ATCC® SCRC-1041™) cultured in DMEM medium (Gibco, Grand Island, NY, USA) supplemented with 2% fetal bovine serum (Gibco), 100 μg/mL streptomycin, and 100 U/mL penicillin. After lysing the cells, the culture supernatant was collected and centrifuged at 500 *g* for 5 min. The cellular debris-free tachyzoites were centrifuged at 1,800 *g* for 15 min and the pellet was resuspended in RPMI 1640 medium (Gibco, Thermo Fisher Scientific, Waltham, MA, United States of America) without supplementation.

### Isolation of PBMCs and Cytokine Quantification

About 15 mL of peripheral blood, which was collected from 30 individuals as described above, was centrifuged as separate samples in a Histopaque 1,077 g/mL (Sigma-Aldricth Produtcs, Merck KGaA, Darmstadt, Germany) gradient. The fraction of mononuclear cells was adjusted to 1 × 10^6^ cells/well, after which the cells were plated in 24-well plates and cultured in RPMI 1640 medium (Gibco) without supplementation at 37°C with 5% CO_2_. The PBMCs were incubated with concanavalin A (10 μg/mL) or infected with *T. gondii* RH, RHΔ*rop16* or RHΔ*rop18* live tachyzoites with a multiplicity of infection (MOI) of 1:3 over a 24 h period. RPMI was used as control. Supernatants were collected and IFN-γ, IL-10, and IL-1β levels were determined using a commercial enzyme-linked immunosorbent assay (Biolegend, San Diego, CA, USA), results were expressed as pg/mL.

### Immune-Related Host Genes Polymorphisms

Single Nucleotide Polymorphisms (SNPs) in the genes encoding the following proteins were evaluated: IL-1β (rs1143634, rs16944, rs1143627); IL-10 (rs1800871), and IFN-γ (rs2430561). Polymorphisms in the purinoreceptor *P2RX7* (rs1718119, rs1621388, rs2230912) and in the Toll-like receptor gene, *TLR-9* (rs352140), were included. Amplification products were analyzed by capillary electrophoresis. The mini-sequencing technique or “ddNTP primer extension” was used as previously reported (Naranjo-Galvis et al., 2018). Briefly, after genomic DNA was isolated from blood cells using the QIAGEN DNA mini kit (QIAGEN), the SNP-containing regions of interest were PCR-amplified using initiation primers. PCRs, which were carried out in 10 μL volumes, contained 1 to 10 ng of genomic DNA, 1X QIAGEN Multiplex PCR Master Mix (QIAGEN N.V., Venlo, The Netherlands), 1X Q-solution and 0.2–0.6 μM of each specific primer. PCR-amplification of the fragments was performed in a Veriti Thermal Cycler (Applied Biosystems, USA). Using this pre-amplification product as template, multiplex reactions for the detection of SNPs were carried out using the mini-sequencing method (SBE, single base extension). The data were analyzed according the color of the peaks and fragment sizes, using the GeneMapper v3.2 software (Applied Biosystems, USA).

### Western Blot Analysis

PBMCs were cultured for 24 h with RH, RHΔ*rop16* or RHΔ*rop18* strains. Then, cells were lysed in RIPA buffer (Amresco, USA) containing a protease inhibitor cocktail (Amresco) and phosphatase inhibitor (Sigma-Aldrich, Darmstadt, Germany). Equivalent amounts of protein were electrophoresed on 10% SDS polyacrylamide gels and then electroblotted onto 0.45 μm nitrocellulose membranes (10600007, GE healthcare). After blocking with 3% milk protein for 30 min at room temperature, the membranes were incubated with the following primary antibodies: anti-phopho-STAT3 (Tyr 705) (Abcam, UK), anti-phopho-STAT6 (Tyr 641) (Abcam), and anti-IL1β (Santa Cruz Biotechnology, USA). The membranes were then washed three times with PBS containing Tween 20 and incubated with polyclonal goat anti-rabbit IgG conjugated with alkaline phosphatase (Sigma-Aldrich, Darmstadt, Germany) for 1 h at room temperature. Positive reactions were detected using Novex® AP Chromogenic Substrate BCIP/NBT (Thermo Fisher, USA). Densitometry analysis was conducted using ImageJ (Schneider et al., 2012), and the signal value from each band was normalized using β-actin (Ambion, USA) as the normalization protein. PBMCs from 3 individuals were randomly selected from each group and the normalized protein expression levels were plotted in a histogram.

### Statistical Analysis

The concentration of each cytokine was expressed in pg/mL. The normality of the data was determined using the Kolmogorov-Smirnov test. The non-parametric Mann–Whitney test was used for comparing the cytokine levels between the different groups (OT, Asym, and Neg). To compare differences in cytokine production under the different stimuli (RH, RHΔ*rop16*, and RHΔ*rop18*) within the groups, non-parametric data were analyzed using Wilcoxon signed-rank test. Bar error represent the mean and standard deviation of each group. Correlation analyses were based on the Spearman coefficient. Statistical significance was defined as *p* < 0.05. All data were analyzed using Prism 6.01 software (GraphPad Software version 6.01, San Diego California).

## Results

### Clinical Characteristics of the Study Subjects

Twenty IgG positive individuals for *T. gondii* (12 OT and 8 Asym), and 10 Neg individuals participated in this study, having a mean age of 32.8 years (range: 23–61 years) and 58% female overall. There were no significant differences in gender or age distribution between groups.

### Lower IFN-γ Production Occurred in the Ocular Toxoplasmosis Group Compared With the Chronic Asymptomatic Group Independently of ROP16 and ROP18

**Figure 1:**
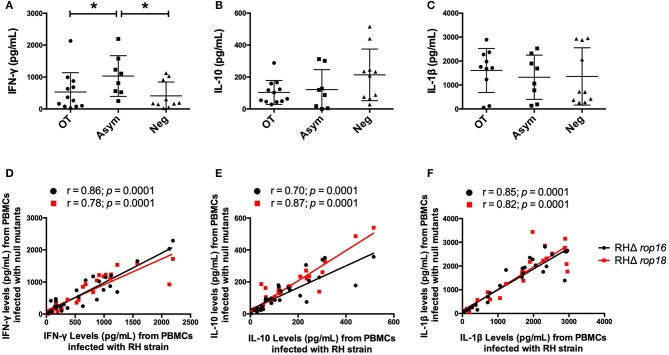
Cytokine secretion by PBMCs infected with the RH strain of *T. gondii*. The concentration of IFN-γ **(A)**, IL-10 **(B)**, and IL-1β **(C)** were determined by ELISA in supernatants at 24 h post-infection (h.p.i.). A correlation analysis on RH, RHΔ*rop*16 and RHΔ*rop*18 strains for each cytokine was performed. The x axis indicates the concentration of IFN-γ **(D)**, IL-10 **(E)**, and IL-1β **(F)** for RH infections. The y axis indicates the same cytokine as in x axes for RHΔ*rop*16 (black circles) or RHΔ*rop*18 (red squares) *T. gondii* infected cells. Each point represents the concentration of cytokines of a single patient. Results **(A–C)** are represented as mean ± SD; *p < 0.05, Mann-Whitney *U* test and the correlations were determined by Spearman's correlation tests. OT, Ocular toxoplasmosis; Asym, Chronic asymptomatic infection; Neg, *T. gondii* seronegative individuals.

We first investigated the cytokines secreted by the PBMCs obtained from individuals with toxoplasmosis (OT and Asym groups) and Neg individuals upon stimulation with WT *T. gondii* RH tachyzoites. IFN-γ production after parasite infection was higher in PBMCs from the Asymp group than in the OT group (*P* = 0.0287) or the Neg group (*P* = 0.0205), indicating that this cytokine tended to be higher in individuals whose infections were resolved and did not have ocular lesions (Figure 1A). In contrast, no significant differences were observed for IL-10 and IL-1β secretion among groups (Figures 1B,C).

**Figure 2:**
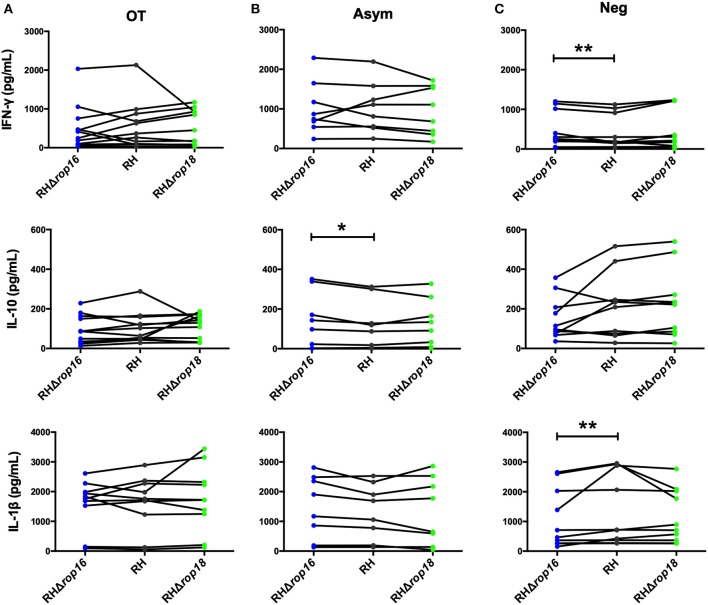
Comparison of cytokine production by PBMCs infected with *T. gondii* RH, RHΔ*rop*16, and RHΔ*rop*18 in culture supernatants at 24 h. OT **(A)**, Asym **(B)**, and Neg **(C)**. *p < 0.05, **p < 0.01; Wilcoxon signed-rank test. Lines are representative of each individual. OT, Ocular toxoplasmosis; Asym, Chronic asymptomatic infection; Neg, *T. gondii* seronegative individuals.

We next investigated whether differences observed in cytokine secretion among groups was related to the *T. gondii* ROP16 and ROP18 virulence factors. First, we confirmed that the mutant parasites indeed lacked the ROP18 protein (Figure S1) or the *rop16* gene (Figure S2). Then, we evaluated the cytokine secretion levels of PBMCs obtained from Asymp, OT, or Neg individuals after infection with live RHΔ*rop16* and RHΔ*rop18* parasite strains. In the group OT, a difference in cytokine production was not observed after *ex-vivo* infection with *rop16* or *rop18* knock-out and WT strains (Figure 2A). However, it is important to note that PBMCs from some individuals clearly responded differently to the KO strains when compared with the WT strain, but this was not related with the status of the infection. Only chronic-asymptomatic individuals (Asym) showed a moderately significant increase in IL-10 production when stimulated with RHΔ*rop16* compared to WT (Figure 2B). In seronegative (Neg) individuals, infection with RHΔ*rop16* strain induced higher levels of IFN-γ compared with WT strain infection. Conversely, the IL-1β concentration was lower when infected with RHΔ*rop16* strain (Figure 2C), showing an opposite relationship between IFN-γ and IL-1β, which are markers for Th1 response. Thus, individuals with OT produced lower levels of IFN-γ than chronic asymptomatic individuals, but this difference seems not to be dependent of the ROP16 and ROP18 *T. gondii* proteins.

We also performed a linear regression analysis with RH, RHΔ*rop16* and RHΔ*rop18*, in order to evaluate the production of each cytokine (Figures 1D–F). The tendency in the levels of each cytokine produced by the PBMCs was similar when infected with each strain. However, a slight correlation was observed between IFN-γ and IL-10 (Figure S3) and no correlation was observed between IL-1β and IL-10 (Figure S4), which indicates that the increase in these inflammatory cytokines was not followed by the regulatory effect of IL-10 in any group of the individuals we evaluated.

### Polymorphisms in the Host's Immune-Related Genes Influence the Cytokine Profile

**Figure 3:**
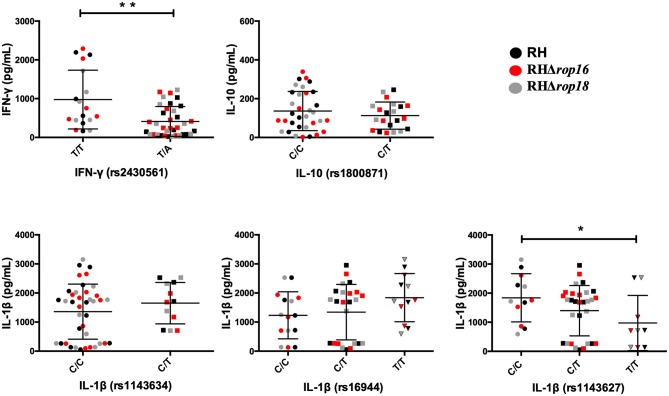
Cytokine production by PBMCs at 24 h post-infection with *T. gondii* RH (Black), RHΔ*rop16* (red), and RHΔ*rop18* (gray) was stratified by genotype of the following SNPs: IFN-γ (rs2430561), IL-10 (rs1800871), and IL-1β (rs1143634, rs16944, rs1143627). The cytokines levels were measured by ELISA. Results are represented by circles or square for individual data and bars are the mean ± SD; *p < 0.05, **p < 0.005. Mann-Whitney *U* test was performed.

As knocking out of *T. gondii rop16* or *rop18* genes did not explain most of the differences observed in the cytokine production between individuals or groups, we decided to evaluate polymorphisms in the aforementioned cytokines and other genes from the host that are immune response related. Therefore, to determine the influence of polymorphisms on the cytokine profile, we evaluated SNPs in encoding genes for *IFN*-γ, *IL-10*, and *IL-1*β in a subgroup of 9 individuals with OT, 4 Asym, and 5 Neg. We also included polymorphisms in encoding genes for the purinoreceptor *P2RX7* and *TLR-9*, these two genes were of interests since polymorphisms in them are related with toxoplasmic retinochoroiditis (Ferrari et al., 1997; Peixoto-Rangel et al., 2009). As no statistical differences among clinical groups were found we decided to perform an analysis assuming all samples in one single group regardless of clinical condition or infecting strain. The IFN-γ, IL-10, and IL-1β levels stratified by the reference SNP (rs) and genotype are shown in Figure 3.

**Figure 4:**
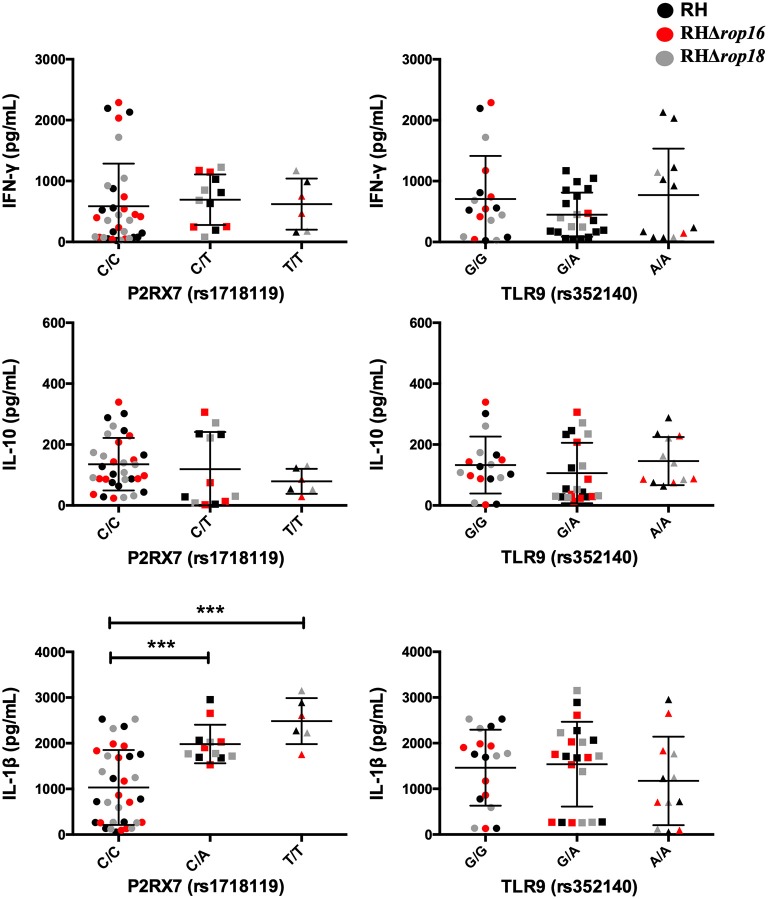
Cytokine production by PBMCs at 24 h post-infection with *T. gondii* RH (Black), RHΔ*rop16* (red), and RHΔ*rop18* (gray) was stratified by genotype of the following SNPs: P2RX7 (rs1718119) and TLR9 (rs352140). The levels of cytokines were measured by ELISA. Results are represented by circles or square for individual data and bars are the mean ± SD; ***p < 0.001. Mann-Whitney *U* test was performed.

The levels of IFN-γ were higher in individuals with T/T genotype when compared with the T/A genotype (656.2 vs. 248 pg/mL, *P* = 0.002). In the same way, a significant difference was observed in IL-1β polymorphism (rs 1143627), where C/C genotype is related to higher production of this cytokine compared with T/T genotype (*P* = 0.033). In contrast, no statistical differences were found for IL-10 levels and polymorphisms. On the other hand, when we evaluated the influence of the genotype of the P2RX7 (rs1718119) membrane receptor on the cytokine profile, we found that the T/T (*P* = 0.0004) and C/A genotype (*P* = 0.0009) are related to IL-1β higher levels than the C/C genotype (Figure 4). Finally, no statistical differences were found between TLR-9 polymorphisms (rs352140; C/C *n* = 2; C/T *n* = 4, T/T *n* = 3) and the cytokines levels we evaluated (IFN-γ mean levels: C/C 715 pg/ml vs. C/T 1402 vs. T/T 1688 pg/ml, *P* = 0.86; IL1-β mean levels: C/C 65 vs. C/T 331 pg/ml vs. T/T 445 pg/ml, *P* = 0.54; IL10 mean levels: C/C 367 pg/ml vs. C/T 692 pg/ml vs. T/T 668 pg/ml, *P* = 0.54).

### STAT3 and STAT6 Phosphorylation Is Not Dependent on the *T. gondii* ROP16 Protein in Human PBMCs

**Figure 5:**
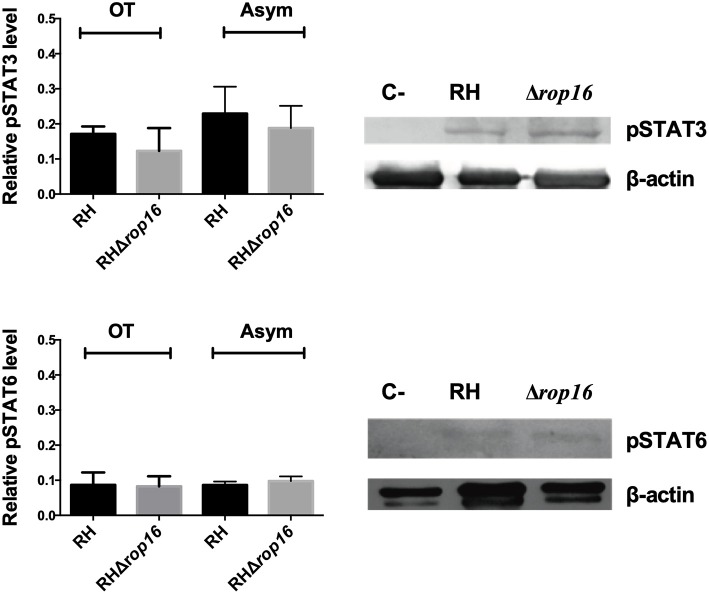
Phosphorylation of STAT3 and STAT6 by ROP16 protein. PBMCs were infected with *T. gondii* RH or RHΔ*rop16* strains for 24 h. STAT phosphorylation was evaluated by Western blot of PBMCs lysates from OT and Asym individuals. The western blot results were quantified by densitometry and Mann-Whitney *U* test was performed with no statistically significant differences (*p* > 0.05) between the stimulus in either of the clinical cases evaluated (TO and Asym). Histogram represent mean of relative levels of protein ± SEM of three independent experiments. One representative western blot result for OT and Asym group is shown. C(-), Uninfected PBMCs were used as a negative control; OT, Ocular toxoplasmosis; Asym, Chronic asymptomatic infection.

The kinase activity of ROP16 has been shown to be essential for downregulating the IL-12 mediated response by phosphorylating STAT3 and STAT6 transcription factors (Saeij et al., 2006, 2007). However, we did not find a significant difference in proinflammatory cytokine levels (IFN-γ and IL-1β) in the OT and Asym groups. To evaluate whether the kinase activity of ROP16 might affect the STAT3 (Tyr 705) and STAT6 (Tyr 641) transcription factors, we infected human PBMCs with live RH or RHΔ*rop16* strains over a 24 h period. The resultant cells were lysed, and their intracellular extracts were analyzed by western blot. In the absence of *rop16* (RHΔ*rop16*), the amount of phosphorylated STAT3 and STAT6 when separately infected with each parasite strain was not significantly different (*P* > 0.05) (Figure 5).

### Immature Pro-IL-1β Levels in Human PBMCs Were Higher in RHΔrop18 Infections

**Figure 6:**
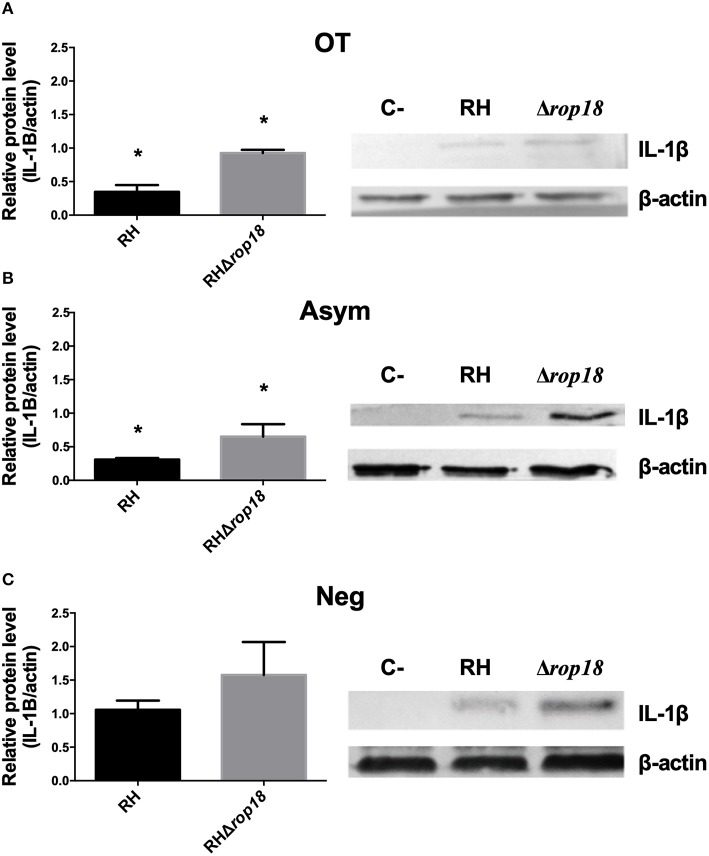
Immature IL-1β in lysates from PBMCs stimulated with *T. gondii* RH and RHΔ*rop*18. The presence of immature pro-IL-1β was evaluated using a western blot and quantified by densitometry. Histogram represent the mean of relative levels of protein ± SEM of three independent experiments. The OT **(A)**, Asym **(B)**, and Neg **(C)** clinical groups are shown. *p < 0.05, Mann-Whitney *U* test. One representative western blot for each clinical group is shown.

It has been reported that the ROP18 virulence factor phosphorylates the dimerization domain of the p65 NF-κB subunit, leading to its downregulation and further reduction of TNF-α, IL-6, and IL-12 secretion in murine and human macrophages cell lines (Du et al., 2014). Because transcription of the IL-1β gene is under NF-κB control, we evaluated the levels of pro-IL-1β in cell lysates of PBMCs infected with either RH or RHΔ*rop18* tachyzoites. We found higher levels of pro-IL-1β in cells infected with RHΔ*rop18* than in cells infected with RH tachyzoites in all groups (OT, Asym, Neg) (Figure 6). Intriguingly, as we showed before in this work, there were no significant difference in the secretion of IL-1β when PBMCs are infected with RH or RHΔ*rop18* strains (Figures 1C, 2), suggesting the existence of a second mechanism influencing the observed differences between the groups in the production of this cytokine.

## Discussion

Cytokines have been shown to play an important role in the pathogenesis of toxoplasmosis (Sullivan and Jeffers, 2012). Following the multiplication phase, where the parasites disseminate throughout the body, the host's immune system takes control and eliminates most of the parasites, mainly by cellular responses such as IFN-γ production driven by Th1 type responses (Pifer and Yarovinsky, 2011). Here we found that individuals with ocular toxoplasmosis produce low levels of IFN-γ compared with the chronic asymptomatic individuals, suggesting that the development of this clinical manifestation (OT) is associated with a defect to produce adequate levels of IFN-γ. This finding is similar to that reported previously, where IFN-γ levels were higher in asymptomatic individuals than in patients with cerebral (Meira et al., 2014) or ocular (De-la-Torre et al., 2013) toxoplasmosis. We also found that the IFN-γ levels were higher in the asymptomatic infected group than in the seronegative group. This finding could be related to the release of high levels of IFN-γ during a chronic infection, by the parasite-specific T lymphocytes, that are required to prevent cyst reactivation (Sarciron and Gherardi, 2000).

A concomitantly produced cytokine that normally acts as a negative feedback mechanism of IFN-γ is IL-10 (Damsker et al., 2010). IL-10 is an immunomodulatory cytokine produced by several cell types (Garra et al., 2004), and in OT Colombian patients it seems to be central in the induction of the permissive state seen in the eye (De-la-Torre et al., 2014; Torres-Morales et al., 2014). However, we did not find any difference in the secretion of this cytokine between groups. One possible explanation lies in the fact that in our previous studies we used total lysate antigen to stimulate PBMCs, but because *T. gondii* release proteins that are involved in the parasite's immune evasion mechanisms in a highly regulated manner (Tosh et al., 2016), the use of live parasites is recommended for this type of immunological experiments (Acosta Davila and Hernandez De Los Rios, 2019).

Our research group has previously suggested that severe ocular infections in South America are caused by highly variable *T. gondii* strains and are characterized by a completely different local immune response pattern and much higher ocular parasite loads (De-la-Torre et al., 2013; Pfaff et al., 2014), where the lower Th1 response in Colombian patients with OT, as compared with European patients, can be explained by a specific modulation of the immune response by South American strains (De-la-Torre et al., 2014). To determine whether this immune response modulation is related to the ROP16 or ROP18 virulence factors, we stimulated the PBMCs obtained from the chronic asymptomatic individuals, patients with OT and *T. gondii* seronegative controls with the knock-out (RHΔ*rop16* and RHΔ*rop18*) parasite strains, separately. Our findings show that ROP16 have a modulatory effect on the production of IFN γ only in seronegative individuals, suggesting that secondary response can overcome the immunoregulatory effect of this virulent protein.

Furthermore, it has been reported that the ROP16 protein phosphorylates host STAT3 and STAT6 transcription factors, which limits the protective Th1 cytokine response (Butcher et al., 2011) and rop16-deficient type I parasites fail to active STAT3/6 (Denkers et al., 2012). In our study, phosphorylation of STAT3 and STAT6 transcription factors after infection with *T. gondii* RH or RHΔ*rop16* was not different between the OT and Asym groups. It is possible that an alternative phosphorylation pathway or pathways are activated during the invasion process, as occurs in other mammalian cells where *T. gondii* activated-signaling mediates ROP16-independent STAT3 activation (Portillo et al., 2017). It is also possible that chronic inflammation can set up altered microenvironments that are encountered by circulating PBMCs, and that abnormal cytokines profiles within these microenvironments could alter the host's signaling pathways (Montag and Lotze, 2006).

The next question we addressed was whether polymorphisms in the host's immune system-related genes were associated with differences in cytokine secretion from the PBMCs infected with *T. gondii*. We investigated this because during toxoplasmosis infection, polymorphisms in both IFN-γ (rs2430561) and IL-10 (rs1800871) and the “GAG” haplotype in the IL-1β gene's promoter (SNPs at rs1143634, rs1143627, rs16944) are associated with the development of OT in the Colombian population (Naranjo-Galvis et al., 2018). Regarding the IFN-γ promoter (SNP rs2430561) polymorphism, we found that individuals with the T/T genotype produced higher levels of IFN-γ than those with T/A alleles. This results are in agreement with the findings from previous studies (De Albuquerque et al., 2009; Neves et al., 2013; Naranjo-Galvis et al., 2018) where the A-allele was found to enhance susceptibility to OT, and also shown the importance of host genetics in terms of IFN-γ secretion in the anti-parasite response. The T/T genotype has previously been reported to be associated with protection against the retinochoroiditis caused by toxoplasmosis (De Albuquerque et al., 2009). In the present study, the T/T genotype and IFN-γ showed no relationship with the clinical condition, but this may have been related to the lower statistical power that came into play with our study.

Finally, it is known that ATP-binding to the purinergic P2X7 (encoded by P2RX7) receptor, stimulates pro-inflammatory cytokines and can lead to killing of intracellular pathogens. Activation of P2X7 stimulates inflammasome activation and secretion of IL-1β (Ferrari et al., 1997). Here, we found that individuals carrying the T-allele in *P2RX7* gene (SNP rs1718119) produced higher levels of IL-1β, which may represent a protective factor against *T. gondii*. This result is in agreement with previous work where the ancestral T-allele (SNP rs1718119) was strongly protective against toxoplasmic retinochroiditis (Jamieson et al., 2010).

In summary, our data show that in PBMCs from individuals with chronic infection (OT and Asym), the production of proinflammatory cytokines such as IFN-γ and IL-1β does not seem to be influenced by ROP16 or ROP18 proteins from *T. gondii*, but by the host's polymorphisms in the cytokine genes. These results indicate that the immune response to the parasite in humans does no only depend on the presence of virulence factors like ROP16 and ROP18 in the parasite, but on the genetic susceptibility of the host to the parasitic infection also.

## Data Availability Statement

The raw data supporting the conclusions of this manuscript will be made available by the authors, without undue reservation, to any qualified researcher.

## Ethics Statement

The studies involving human participants were reviewed and approved by Comité de Bioética, Universidad del Quindío. The patients/participants provided their written informed consent to participate in this study.

## Author Contributions

AH designed and performed the experiments and wrote the paper. MM-L, LM-M, AA, MV-M, and NC performed experiments. AT recruited and examined the OT patients. JS-A designed experiments. JG-M designed experiments and analyzed the data.

### Conflict of Interest

The authors declare that the research was conducted in the absence of any commercial or financial relationships that could be construed as a potential conflict of interest.

## Supplementary Material

Figure S1 Verification of the absence of ROP18 protein in the total antigen extracts by western blot. (1) Molecular weight markers. (2) *T. gondii* RH strain. (3) *T. gondii* KO-*rop18* strain.

Figure S2 Verification of absence of *rop16* gene after specific PCR in a KO strain. (1) Molecular marker. (2) *T. gondii* RH strain. (3) *T. gondii* KO-*rop16* strain.

Figure S3 Linear regression analysis on RH, RHΔ*rop*16 and RHΔ*rop*18 strains between IFN-γ and IL-10, showing no correlation between these cytokines.

Figure S4 Linear regression analysis on RH, RHΔ*rop*16 and RHΔ*rop*18 strains between IL-1β and IL-10, showing no correlation between these cytokines.
