# Supplementary figures and images for: Influence of Two Major Toxoplasma Gondii Virulence Factors (ROP16 and ROP18) on the Immune Response of Peripheral Blood Mononuclear Cells to Human Toxoplasmosis Infection
Source: Front Cell Infect Microbiol. 2019 Dec 4;9:413. doi: 10.3389/fcimb.2019.00413 (PMC6904310; doi:10.3389/fcimb.2019.00413)

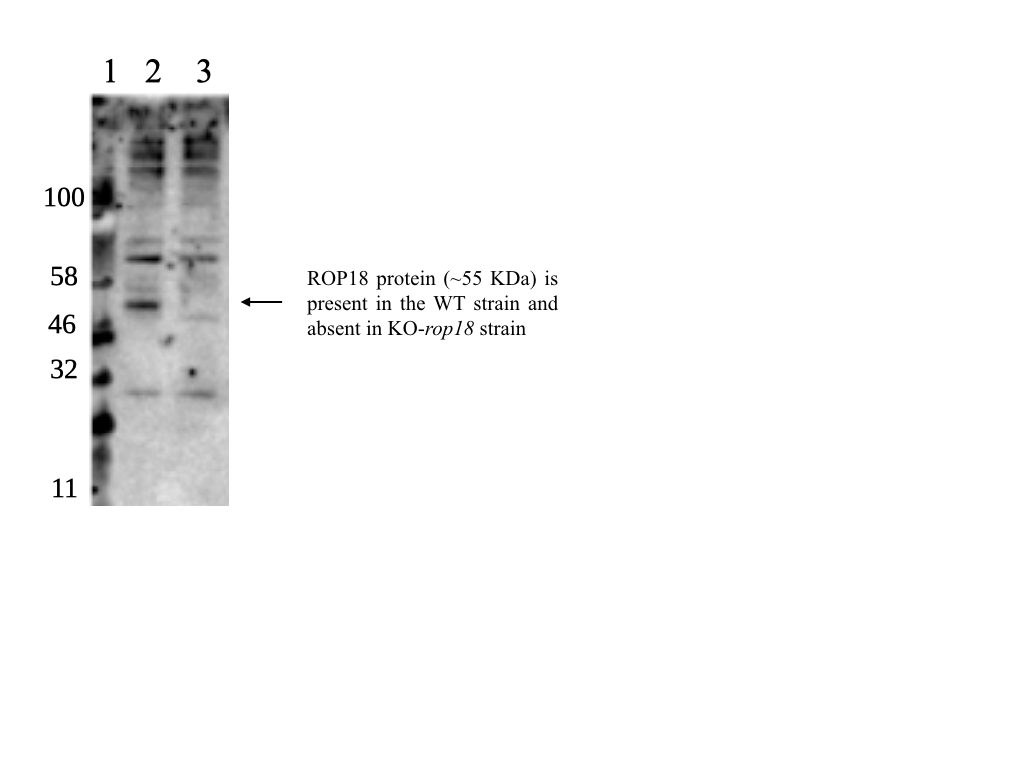

Supplement: Figure S1 — Verification of the absence of ROP18 protein in the total antigen extracts by western blot. (1) Molecular weight markers. (2) T. gondii RH strain. (3) T. gondii KO-rop18 strain. [file Image_1.TIFF]

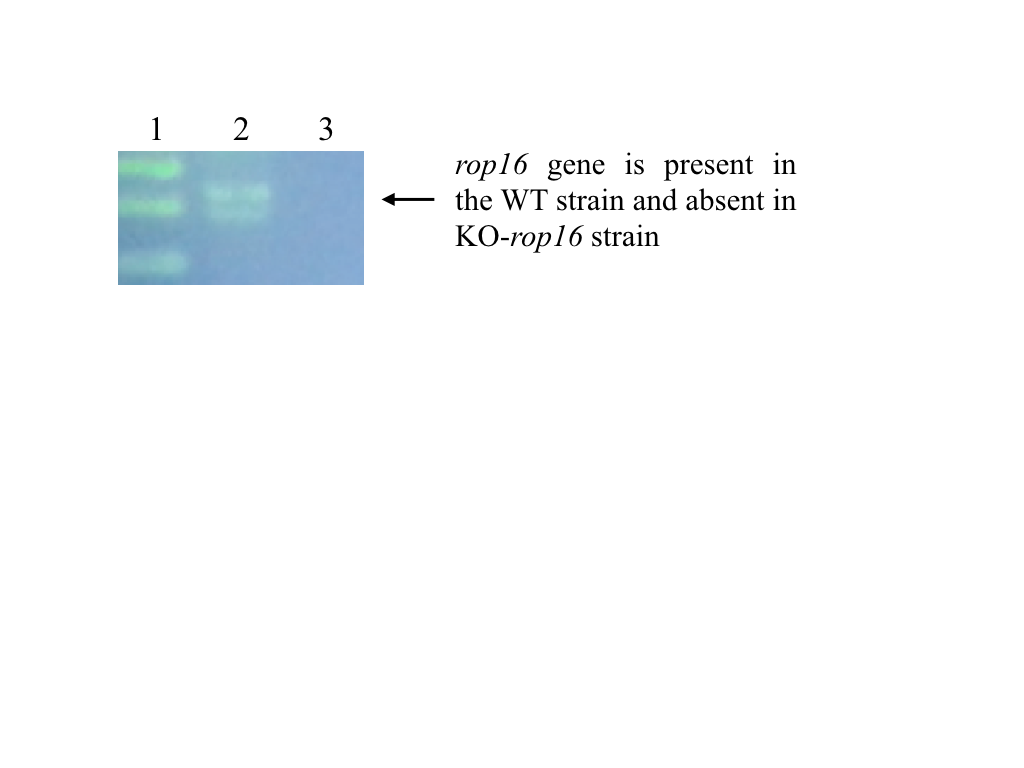

Supplement: Figure S2 — Verification of absence of rop16 gene after specific PCR in a KO strain. (1) Molecular marker. (2) T. gondii RH strain. (3) T. gondii KO-rop16 strain. [file Image_2.TIFF]

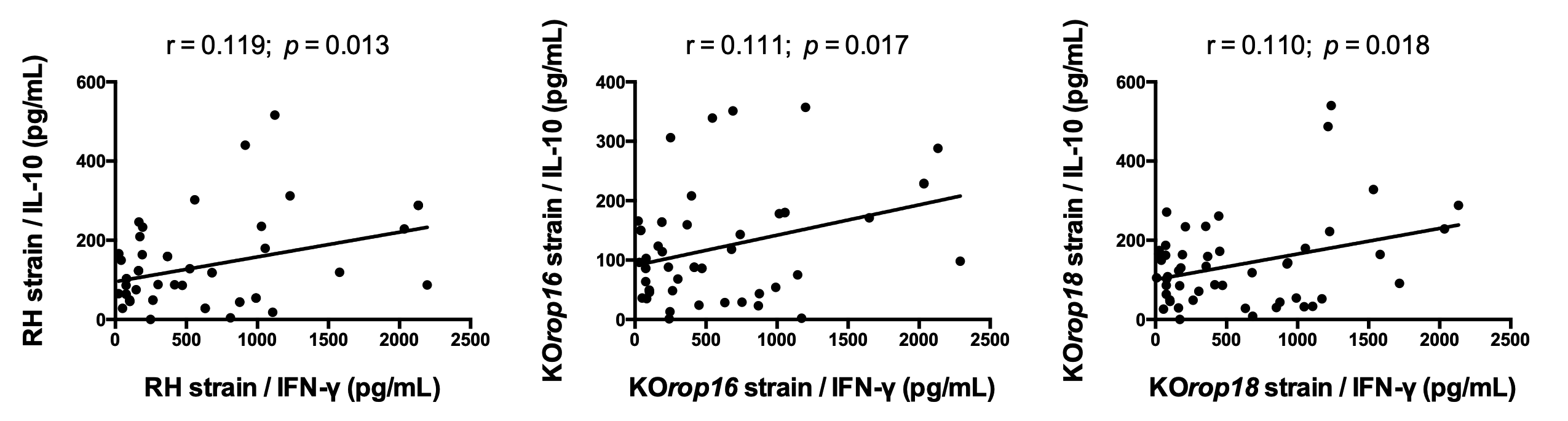

Supplement: Figure S3 — Linear regression analysis on RH, RHΔrop16 and RHΔrop18 strains between IFN-γ and IL-10, showing no correlation between these cytokines. [file Image_3.TIFF]

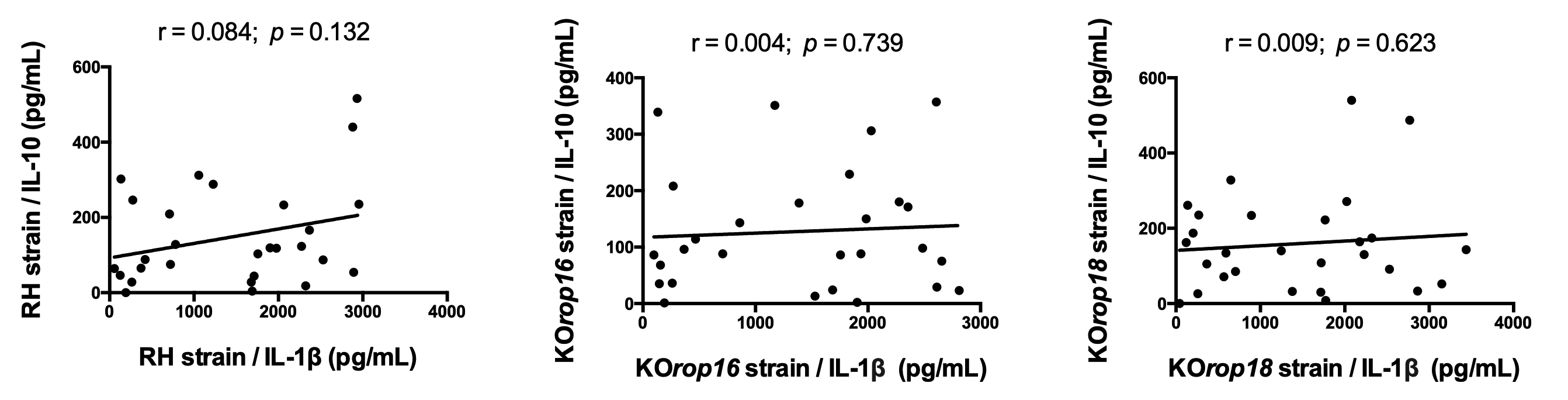

Supplement: Figure S4 — Linear regression analysis on RH, RHΔrop16 and RHΔrop18 strains between IL-1β and IL-10, showing no correlation between these cytokines. [file Image_4.TIFF]
